# Psychotherapeutic drug‐induced life‐threatening arrhythmias: A retrospective analysis using the Japanese adverse drug event report database

**DOI:** 10.1002/joa3.12936

**Published:** 2023-10-03

**Authors:** Saki Yokohara, Masayuki Hashiguchi, Tsuyoshi Shiga

**Affiliations:** ^1^ The Jikei University School of Medicine Tokyo Japan; ^2^ Department of Clinical Pharmacology and Therapeutics The Jikei University School of Medicine Tokyo Japan

**Keywords:** Japan, psychotherapeutic drug, QT prolongation, spontaneous reporting system, ventricular arrhythmia

## Abstract

**Background:**

Drug‐induced life‐threatening ventricular arrhythmias including torsade de pointes (TdP), ventricular tachycardia (VT), and ventricular fibrillation (VF) are serious cardiac side effects. Psychotherapeutic drugs are known to be risk factors for arrhythmias. The aim of this study was to evaluate psychotherapeutic drugs associated with life‐threatening ventricular arrhythmias using the Japanese Adverse Drug Event Report (JADER) database.

**Methods:**

From the JADER database (April 2004 to September 2022), cases of TdP, VT, VF, and QT prolongation in patients taking psychotherapeutic drugs as ‘suspected drugs’ were extracted. The adjusted reported odds ratio (aROR) was calculated to identify potential drugs involved in combined TdP/VF/VT or combined QT prolongation/TdP.

**Results:**

Of the 4,530,772 cases reported, life‐threatening arrhythmia‐related adverse events were reported in 1760 cases (QT prolongation 1261, TdP 192, VF 108, VT 199) among 909 patients; 58.9% of patients were female, and the highest incidence was among patients aged 80–89 years (18.6%), followed by patients aged 70–79 years (15.4%). The highest aROR for TdP/VF/VT was found for trazodone (17.1), followed by sulpiride (10.8), haloperidol (9.8), donepezil (9.1), and fluvoxamine (7.9). The highest aROR for QT prolongation/TdP was found for guanfacine (87.8), followed by sultopride (60.1), escitalopram (21.0), trazodone (12.8), and donepezil (9.3).

**Conclusions:**

This study showed that typical antipsychotics, antidepressants, and antidementia drugs were associated with life‐threatening arrhythmia‐related adverse events in a Japanese clinical setting. These events were more frequent in women and elderly individuals.

## Graphical abstract

The JADER database showed that antipsychotics, antidepressants, and antidementia drugs were associated with life‐threatening arrhythmia‐related adverse events in a Japanese clinical setting. These events were more frequent in patients aged 70–89 years.

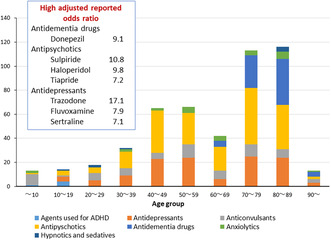

## 1 INTRODUCTION

Drug‐induced life‐threatening ventricular arrhythmias, including torsade de pointes (TdP) associated with QT prolongation, are serious cardiac side effects.^1^ Sudden cardiac death (SCD) is one of the leading causes of early mortality among psychiatric patients.^2^ Psychiatric patients sometimes take antidepressant or antipsychotic medications, and drug‐induced arrhythmias are partly responsible for the development of SCD.^3^, ^4^ A nationwide cohort study (Danish Cardiac Arrest Register) reported that treatment with any antipsychotic drug was associated with out‐of‐hospital cardiac arrest.^5^ Another nationwide cohort study (Taiwan's National Health Insurance Research Database) reported that the use of antipsychotic drugs was associated with an increased risk of ventricular arrhythmias and/or SCD.^6^

Several antipsychotics and antidepressants on the market are known to cause QT prolongation. Central nervous system drugs, including psychotropic and antidepressant drugs, have been noted to have the second highest frequency of QT prolongation/TdP in spontaneous adverse event reports after cardiac drugs.^7^ Many psychotherapeutic drugs that have a unique site of action have been developed, but it is difficult to detect whether a drug induced QT prolongation/TdP in preclinical or preapproval clinical trials, although the International Conference on Harmonization (ICH) E14 guidelines advocate thorough QT/QTc studies to investigate how drugs under development affect the QT interval. In particular, rare adverse events (AEs) have been detected in the postmarketing phase in a real‐world database. Recently, from the Japanese Adverse Drug Event Report (JADER) database, spontaneous AE reporting data showed that QT prolongation/TdP was most frequently reported for cardiovascular drugs, followed by psychotherapeutic drugs.^8^

The aim of this study was to evaluate psychotherapeutic drugs associated with life‐threatening ventricular arrhythmias such as TdP, ventricular tachycardia (VT) and ventricular fibrillation (VF) using the JADER database, a real‐world database in Japan.

## 2 METHODS

### 2.1 Data sources

Information from the JADER database between April 2004 and September 2022 was used in this study. The JADER database compiles domestic AE reports provided to the Pharmaceuticals and Medical Devices Agency (PMDA) by manufacturers and medical institutions and consists of the following four data tables^9^: patient demographic information (DEMO), drug information (DRUG), adverse event information (REAC), and primary disease information (HIST). The DEMO table specifies the sex and age of the patients. The DRUG table provides the drug name, dose, reason for use, and administration duration. The REAC table provides AEs, the results, and the dates of AE occurrence. The HIST table provides information on a patient's underlying diseases.

### 2.2 Data extraction and analysis

The DRUG table describes the involvement of the drug, classifying all drugs as ‘suspected drugs’, ‘interacting drugs’, or ‘concomitant drugs’. In this study, only ‘suspected drugs’ were analyzed. We counted the number of occurrences of TdP, VT, VF, and QT prolongation from all the drugs reported with the same ID. ‘Sudden deaths’ were not counted because these deaths could not be confirmed as arrhythmia‐related. In the JADER database, an AE is coded according to the terminology of preferred terms (PTs) in the Japanese version 25.0 of the ICH Medical Dictionary for Regulatory Activities Japanese Maintenance Organization (MedDRA/J V25.0).

A standardized MedDRA query (SMQ) was used to identify cases of QT prolongation and TdP: ‘Torsade de pointes/QT prolongation (SMQ code: 20000001)’. PT was used to identify ventricular fibrillation (10047290) and ventricular tachycardia (10047302). A search was performed in the JADER database for all AEs mapped to ‘Torsade de Pointes’, ‘ventricular fibrillation’, ‘ventricular tachycardia’, ‘Electrocardiographic QT Prolongation’, and ‘QT prolongation’ from April 2004 to September 2022. After removing duplicate data, only suspected cases of TdP, VT, VF, and QT prolongation were extracted.

### 2.3 Medication definitions

In this study, psychotherapeutic drugs were defined according to the World Health Organization Anatomical Therapeutic Chemical classification as follows: antipsychotics, anxiolytics, hypnotics and sedatives, antidepressants, agents used for attention‐deficit/hyperactivity disorder (ADHD), antidementia drugs and antiepileptics. Psychostimulants and nootropics were excluded from this analysis.

### 2.4 Analysis

Disproportionality analysis was performed by case–noncase methods based on the reporting odds ratio (ROR) and its 95% confidence interval (CI).^10^ The ROR was calculated from the whole database. ‘Cases’ were defined as the reported suspected cases of life‐threatening ventricular arrhythmias (combined TdP, VT, and VF) or QT prolongation/TdP (combined QT prolongation and TdP), while ‘noncases’ were defined as other reported AEs. The RORs were calculated by univariate logistic regression analysis with each occurrence of TdP/VT/VF or QT prolongation/TdP as the objective variable and the use of the drug as the explanatory variable. The adjusted ROR was calculated by multivariate logistic regression analysis using sex, age, and underlying disease (Table S1) as potential confounding variables for the relationship between TdP/VT/VF or QT prolongation/TdP and psychotherapeutic drugs. A signal was considered present when the lower limit of the 95% confidence interval of the ROR exceeded 1. Because it is difficult to compare drugs with very few reports, only drugs with a total of 5 or more reports and an ROR ≥2 for TdP/VT/VF or QT prolongation/TdP were included in the analysis.

An event was determined to be an overdose if the daily dose exceeded the recommendation listed on the dosage and administration package insert of each drug. All missing values and blank data were labeled as ‘not specified’. The distribution of the general characteristics of patients for each item was evaluated by Pearson's chi‐square test. ‘Not specified’ data were excluded from the statistical tests. Analyses were conducted using JMP® 16.2.0 (SAS Institute Inc.) and SAS 9.4 (SAS Institute Inc.).

## 3 RESULTS

Of the 4,530,772 total data entries reported in the JADER database from April 2004 to September 2022, 192 TdP, 199 VT, 108 VF, and 1261 QT prolongation cases were related to 80 psychotherapeutic drugs. These drugs were extracted as ‘suspected drugs’.

Table 1 shows the general characteristics of the patients included in the dataset for the suspected drugs. A total of 58.9% of the patients were females. The sex of 4.8% of the patients was unknown. The age of the patients ranged from newborn to over 100 years. The highest occurrence was in the 80‐ to 89‐year‐old age group (18.6%), followed by the 70‐ to 79‐year‐old age group (15.4%) and the 50‐ to 59‐year‐old age group (12.1%). In the JADER database, there were patients whose ages were listed as ‘infants’, ‘youth’, or ‘elderly’ instead of an age group. Since these patients could not be incorporated into an exact age group, they were tabulated as ‘infant’, ‘youth’, and ‘elderly’ as described.

**TABLE 1:** General characteristics of patients included in the dataset for the suspected drugs.

	*n*	%	*p* value
No. of patients	909		
No. of reported AEs	1760		
Prolonged QT Syndrome/QT Prolongation	1261	71.6	
Torsade de pointes (TdP)	192	10.9	
Ventricular fibrillation (VF)	108	6.1	
Ventricular tachycardia (VT)	199	11.3	<.0001
Sex			
Female	535	58.9	
Male	330	36.3	<0.0001
Not specified	44	4.8	
Age, years			
<10	29	3.2	
≥10 to <20	77	8.5	
≥20 to <30	45	5.0	
≥30 to <40	76	8.4	
≥40 to <50	87	9.6	
≥50 to <60	110	12.1	
≥60 to <70	86	9.5	
≥70 to <80	140	15.4	
≥80 to <90	169	18.6	
≥90	19	2.1	<.0001
Infant	7	0.8	
Youth	4	0.4	
Adult	10	1.1	
Elderly	9	1.0	
Not specified	41	4.5	
Dosage information			
Standard dose	1184	67.3	
Overdose	82	4.7	<.0001
Not specified	494	28.0	
Treatment for AEs			
Discontinuation	847	48.1	
Dose reduction	155	8.8	
Dose escalation	65	3.7	
No change in dosage	185	10.5	<.0001
Not specified	508	28.9	
Outcome			
Death	44	2.5	
Serious (sequelae, unrecovered)	133	7.6	
Non‐Serious	1262	71.7	<.0001
Not specified	321	18.2	

*Note*: The *p* values are from Pearson's chi‐square test. ‘Not specified’ was excluded for the tests.

Infant, Youth, Adult, and Elderly were also excluded from the age group analysis.

Abbreviation: AE, adverse drug event.

Regarding the dose, 4.7% of cases were deemed to be overdoses, which we considered to be relatively low. Target AEs were more common in cases with standard doses (67.3%) and not specified doses (28.0%). When AEs occurred, dose discontinuation (48.7%) was common, while dose reduction was infrequent (8.8%); some AEs led to no dose changes (10.5%) or even led to dose escalation (3.7%). Approximately 71.7% of these cases were nonserious, resolving in recovery or mild resolution. Serious outcomes included sequelae in 0.8% of cases, no recovery in 6.8%, and death in 2.5%. Note that these deaths did not include those categorized as sudden deaths but were those in which ventricular arrhythmia was confirmed and resulted in death.

Table 2 shows the types of reports and reporters of the cases included in the dataset for the suspected drugs. A total of 84.2% of the AE reports were spontaneous reports by health care professionals, and 12.4% were reported in clinical trials. The reporters were mainly physicians, pharmacists, and other health care professionals (89.8%). The vast majority of these were reported by physicians.

**TABLE 2:** Types of reports and reporters of the cases included in the dataset for the suspected drugs.

	*n*	%	*p* value
Type of reports			
Spontaneous report	1482	84.2	
Clinical trial	218	12.4	
Other	58	3.3	<.0001
Not specified	2	0.1	
Type of reporters			
Physician	1255	71.3	
Pharmacist	155	8.8	
Physician/Pharmacist	112	6.4	
Physician/Pharmacist/Other health care professional	9	0.5	
Physician/Other health care professional	16	0.9	
Other health care professional	33	1.9	
Physician/Pharmacist/Consumer, etc.	7	0.4	
Physician/Other health care professional/Consumer, etc.	3	0.2	
Physician/Consumer, etc.	38	2.2	
Pharmacist/Consumer, etc.	20	1.1	
Consumers, etc.	8	0.5	
Pharmacist/Unknown	2	0.1	<.0001
Not specified	102	5.8	

*Note*: The *p* values are from Pearson's chi‐square test. ‘Not specified’ was excluded for the tests.

**FIGURE 1:**
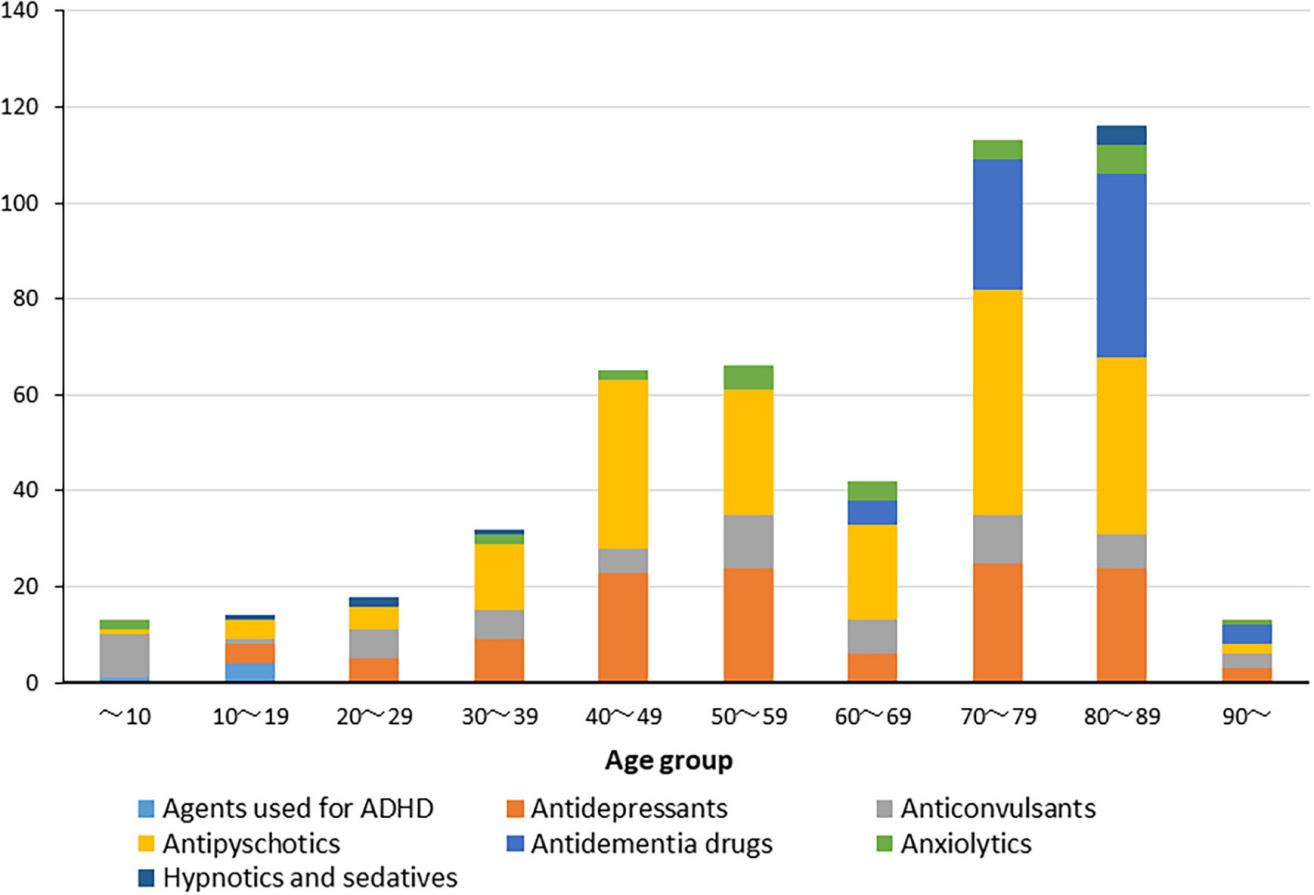
TdP/VT/VF occurrence and drug groups by age group (10 age groups). ADHD, attention‐deficit/hyperactivity disorder.

Table 3 ranks psychotherapeutic drugs associated with TdP/VT/VF according to their adjusted RORs. The highest adjusted RORs were observed for trazodone (antidepressant, serotonin antagonist, and reuptake inhibitor), followed by sulpiride (typical antipsychotic, in some cases classified as an atypical antipsychotic), donepezil (antidementia drug, acetylcholinesterase inhibitor), haloperidol (typical antipsychotic), fluvoxamine (antidepressant, selective serotonin reuptake inhibitor [SSRI]), tiapride (typical antipsychotic), and sertraline (antidepressant, SSRI). The incidence of TdP/VT/VF was higher among patients aged 70–89 years. Additionally, in this age group, patients taking antidementia and antipsychotic medications had higher rates of TdP/VT/VF. (Figure 1).

**TABLE 3:** Psychotherapeutic drugs associated with TdP/VF/VT.

Drugs^a^	Case	Noncase	ROR (95%CI)	Adjusted ROR (95%CI)	PI
Antipsychotics					
Sulpiride	34	2283	9.9 (7.1–13.9)	10.8 (7.7–15.2)	Y
Haloperidol	29	2599	7.4 (5.1–10.7)	9.8 (6.8–14.1)	Y
Tiapride	8	614	8.6 (4.3–17.3)	7.2 (3.6–14.6)	Y
Chlorpromazine	15	2043	4.9 (2.9–8.1)	6.5 (3.9–10.9)	Y
Levomepromazine	7	1542	3.0 (1.4–6.3)	4.3 (2.1–9.1)	Y
Risperidone	23	6756	2.3 (1.4–3.4)	2.8 (1.9–4.3)	N
Antidepressants					
Trazodone	19	802	15.7 (9.9–24.8)	17.1 (10.8–27.1)	Y
Fluvoxamine	17	1860	6.0 (3.7–9.8)	7.9 (4.9–12.8)	Y
Sertraline	23	2362	6.5 (4.3–9.7)	7.1 (4.7–10.8)	Y
Escitalopram	6	950	4.2 (1.9–9.3)	5.0 (2.2–11.2)	Y
Anxiolytics					
Alprazolam	8	1487	3.5 (1.8–7.1)	4.6 (2.3–9.3)	N
Diazepam	6	1305	3.0 (1.4–6.8)	3.6 (1.6–8.0)	N
Antidementia drugs					
Donepezil	65	3382	12.9 (10.1–16.5)	9.1 (7.1–11.7)	Y
Anticonvulsants					
Midazolam	9	2348	2.5 (1.3–4.9)	3.0 (1.6–5.8)	Y

Abbreviations: Adjusted ROR, ROR adjusted by age and sex; CI, confidence interval, N, No; PI, described QT prolongation of package insert; ROR, reporting odds ratio; TdP, torsade de pointes; VF, ventricular fibrillation; VT, ventricular tachycardia; Y, Yes.

^a^ Case≥5 and ROR≥2.

**FIGURE 2:**
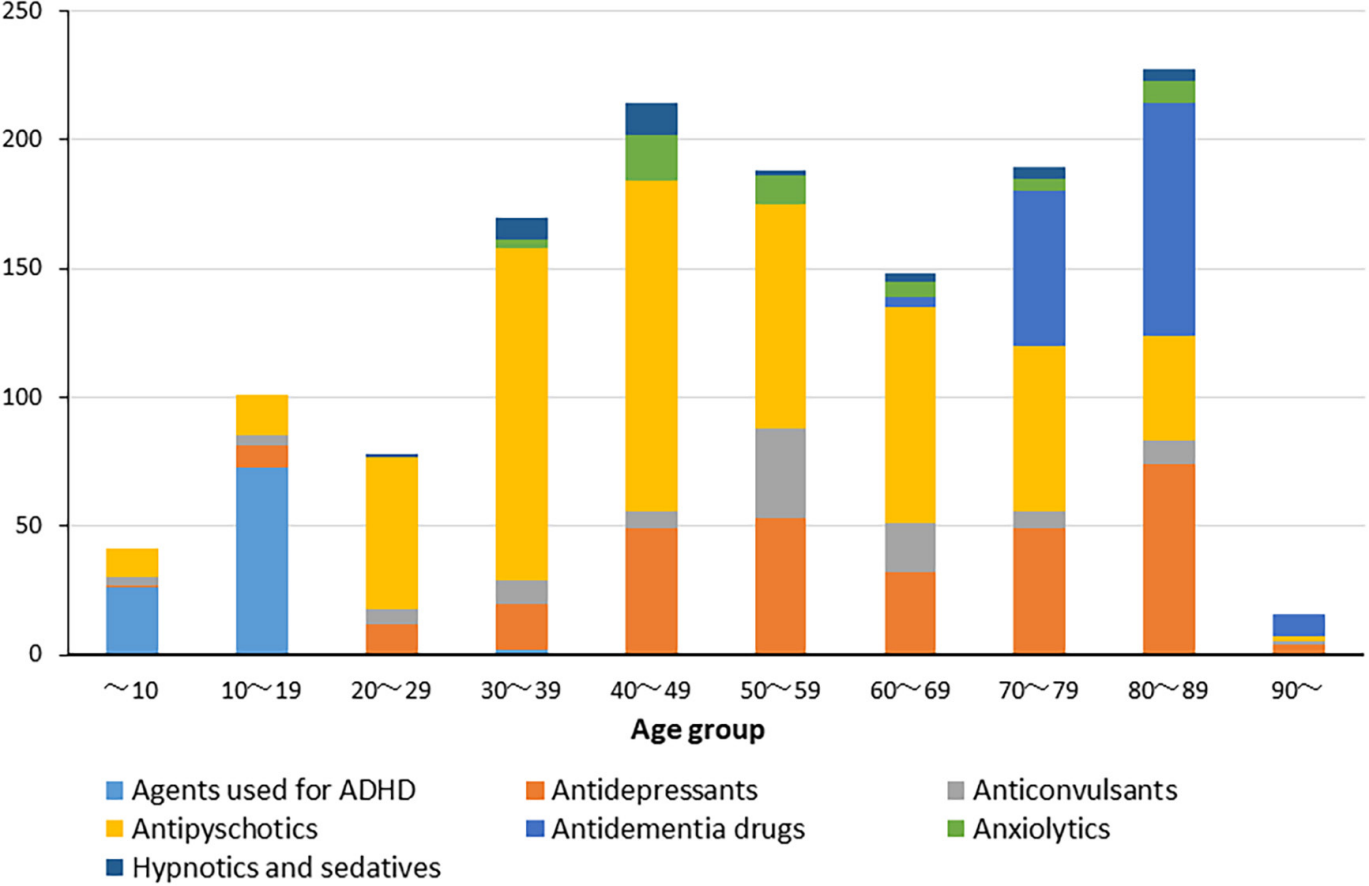
QT prolongation/TdP occurrence and drug groups by age group (10 age groups). ADHD, attention‐deficit/hyperactivity disorder.

Table 4 ranks the psychotherapeutic drugs associated with QT prolongation/TdP according to their adjusted RORs. The highest adjusted RORs were observed for guanfacine (agent used for ADHD), followed by sultopride (typical antipsychotic), escitalopram (antidepressant, SSRI), trazodone (antidepressant), donepezil (antidementia drug), and sulpiride (typical antipsychotic). The incidence of QT prolongation/TdP was low among younger patients but was constant from middle age through old age. Among younger patients, agents were used mainly for ADHD, and among middle‐aged patients, antipsychotics were the most frequent drugs, followed by antidepressants. The usage of antidementia drugs increased among elderly patients. (Figure 2).

**TABLE 4:** Psychotherapeutic drugs associated with QT prolongation/TdP.

Drugs^a^	Case	Noncase	ROR (95%CI)	Adjusted ROR (95%CI)	PI
Agents used for ADHD					
Guanfacine	104	591	58.7 (47.6–72.4)	87.8 (70.7–109.0)	Y
Methylphenidate	17	1493	3.7 (47.6–72.4)	5.0 (3.1–8.1)	N
Antipsychotics					
Sultopride	17	117	47.7 (28.7–79.4)	60.1 (36.0–100.2)	Y
Sulpiride	61	2256	8.9 (6.9–11.5)	9.1 (7.1–11.8)	Y
Haloperidol	47	2581	6.0 (4.5–8.0)	6.9 (5.2–9.2)	Y
Tiapride	13	609	7.0 (4.0–12.1)	6.4 (3.7–11.0)	Y
Paliperidone	54	3595	5.0 (3.8–6.5)	5.7 (4.4–7.5)	Y
Blonanserin	19	1471	4.2 (2.7–6.7)	4.7 (3.0–7.4)	Y
Clozapine	171	16,743	3.4 (2.9–4.0)	4.2 (3.6–4.9)	Y
Levomepromazine	15	1534	3.2 (1.9–5.3)	3.8 (2.3–6.4)	Y
Risperidone	67	6712	3.3 (2.6–4.2)	3.7 (2.9–4.7)	Y
Chlorpromazine	19	2039	3.1 (1.9–4.8)	3.6 (2.3–5.6)	Y
Aripiprazole	66	8027	2.7 (2.1–3.5)	3.1 (2.4–3.9)	Y
Olanzapine	37	4835	2.5 (1.8–3.5)	2.9 (2.1–4.0)	Y
Quetiapine	32	4829	2.2 (1.5–3.1)	2.3 (1.6–3.3)	Y
Antidepressants					
Escitalopram	54	902	19.8 (15.0–26.0)	21.0 (15.9–27.7)	Y
Trazodone	30	791	12.5 (8.7–18.0)	12.8 (8.9–18.5)	Y
Mianserin	10	503	6.5 (3.5–12.2)	6.6 (3.5–12.4)	Y
Sertraline	45	2310	6.3 (4.7–8.5)	6.5 (4.8–8.7)	Y
Fluvoxamine	29	1848	5.2 (3.6–7.4)	5.7 (4.0–8.3)	N
Duloxetine	47	2804	5.5 (4.1–7.4)	5.3 (4.0–7.1)	N
Venlafaxine	23	1883	4.0 (2.7–6.1)	4.3 (2.9–6.6)	Y
Amitriptyline	6	554	3.6 (1.6–8.0)	3.6 (1.6–8.1)	Y
Paroxetine	43	5756	2.5 (1.8–3.3)	2.6 (2.0–3.6)	Y
Anxiolytics					
Alprazolam	18	1477	4.0 (2.5–6.4)	4.6 (2.3–9.3)	N
Diazepam	11	1300	2.8 (1.5–5.0)	3.0 (1.7–5.5)	N
Antidementia drugs					
Donepezil	112	3335	11.2 (9.2–13.5)	9.3 (7.7–11.2)	Y
Rivastigmine	31	2306	4.4 (3.1–6.3)	3.8 (2.6–5.4)	Y
Anticonvulsants					
Levetiracetam	38	4957	2.5 (1.8–3.5)	2.9 (2.1–4.0)	Y
Hypnotics and sedatives					
Flunitrazepam	19	2359	2.6 (1.7–4.2)	2.9 (1.9–4.6)	N

Abbreviations: ADHD, attention deficit/hyperactivity disorder; Adjusted ROR, ROR adjusted by age and sex; CI, confidence interval; N, No; PI, described QT prolongation of package insert; ROR, reporting odds ratio; TdP, torsade de pointes; Y, Yes.

^a^ Case ≥5 and ROR ≥2.

## 4 DISCUSSION

Our study found the following: (1) Cases among females were more common, and the incidence of TdP/VT/VF was high only among elderly patients, although there were a number of reports of QT prolongation/TdP among middle‐aged and elderly patients. (2) Elderly patients taking antidementia medications had a higher incidence of events. (3) The psychotherapeutic drug with highest adjusted ROR for TdP/VF/VT was trazodone, followed by sulpiride, donepezil, haloperidol, fluvoxamine and tiapride, and sertraline. (4) The psychotherapeutic drug with highest adjusted RORs for QT prolongation/TdP was guanfacine, followed by sultopride, escitalopram, trazodone, donepezil, and sulpiride.

### 4.1 Age and sex

The higher incidence of female sex may be related to the older age of the reported patients. In this study, half (55.6%) of the reported patients were 50–89 years old. Women have a higher risk than men of drug‐induced QT prolongation/TdP.^11^ In addition, the high number of reported cases caused by antidementia drugs among patients aged 70 years or older may reflect the reality of Japan's aging society.^12^

Advanced age is also an exacerbating factor for TdP caused by drugs that have a risk of QT prolongation.^13^ A recent large Swedish cohort study showed that the incidence of drug‐induced TdP was higher among elderly people than among young people.^14^ Among elderly individuals, the 70–89 age group was the most frequently reported in the JADER database. The highest frequency of drug use in this age group in Japan may explain these findings.

### 4.2 Peculiarities in Japan

In this study, trazodone, sulpiride, and donepezil had high RORs for life‐threatening ventricular arrhythmias. These three drugs were also associated with QT prolongation/TdP, and QT prolongation is strongly suspected as a major cause of life‐threatening ventricular arrhythmias. Trazodone, a serotonin antagonist and reuptake inhibitor, is used only in Japan and Belgium and is a prescribed antidepressant as well as hypnotic drug in Japan. Although trazodone did not significantly prolong QTc in a thorough QT/QTc study,^15^ a retrospective case analysis of data from a poison center found that trazodone was associated with QT prolongation (QTc >500 ms).^16^ There have also been case reports of QT prolongation associated with trazodone overdose.^17^, ^18^, ^19^ Sulpiride is a typical psychotropic that was approved in Japan in the 1970s but has not been approved in the United States. Sulpiride is a frequently prescribed antipsychotic in Japan^20^ and is widely prescribed by general physicians because it is also indicated for depression and gastroduodenal ulcers. Sulpiride is often prescribed for elderly patients who have physiologically impaired renal function. The urinary excretion rate of the unchanged form of sulpiride is as high as 90%,^21^ which may be associated with increased blood levels of sulpiride in these patients, leading to the development of AEs. Donepezil, an acetylcholinesterase inhibitor, is the most commonly used antidementia drug in Japan.^22^ In Japan, a high prescription rate of antidementia drugs has been reported among elderly individuals, especially among those aged 85 years and older.^23^ A case–control study reported that Japanese patients (mean age 84 years) taking donepezil had significantly longer QT than those not taking the drug.^24^ This is a situation unique to Japan that may be related to the higher risk of age‐related AEs reported among 80–89‐year‐olds in our study.

### 4.3 QT prolongation and ventricular arrhythmias

Guanfacine, a centrally acting α2A adrenergic receptor agonist, is an agent used for ADHD and is a well‐known QT prolonging drug that acts in a dose‐dependent manner.^25^ Although a number of reports about QT prolongation with guanfacine have been mentioned in the JADER database, it is used mostly in young patients, and reports of life‐threatening ventricular arrhythmias are rare in the young age groups in the JADER database. There are few reports of TdP or other ventricular arrhythmias with guanfacine.^26^ One reason for this might be that guanfacine causes a stabilizing effect on cardiac restitution.^27^ Escitalopram was associated with a risk of QT prolongation in a thorough QT/QTc study^28^, ^29^; however, there were few reports of life‐threatening ventricular arrhythmias with escitalopram.^30^ Exposure to known TdP‐risk drugs commonly prolongs QTc on electrocardiography, but life‐threatening arrhythmias are rare.^16^ QT/QTc is now generally accepted as a risk marker for arrhythmias, but it is difficult to predict the risk associated with QT prolongation, such as the development of TdP or SCD.

### 4.4 Psychotherapeutic drug use and cardiovascular risk

In our study, the aROR of life‐threatening ventricular arrhythmias was higher with typical antipsychotics. This is similar to the findings of a Danish study that reported that the use of typical antipsychotics, especially haloperidol, was associated with out‐of‐hospital cardiac arrest, whereas the use of atypical antipsychotics was not.^5^ On the other hand, fluvoxamine and sertraline were associated with a higher aROR of life‐threatening ventricular arrhythmias, but previous reports did not support this result.^31^, ^32^ The reason for this discrepancy may lie in the relationship between depression and cardiovascular disease: depression is a known risk factor for adverse cardiovascular outcomes among patients with cardiovascular disease, and psychosocial and environmental factors are also associated with depression and cardiovascular outcomes.^33^ Most importantly, the majority of patients treated with psychotropic medications are elderly individuals.^34^ In elderly individuals, coexisting cardiovascular disease and other comorbidities or the use of medications that treat or interact with these conditions further increase the risk of life‐threatening ventricular arrhythmias.

### 4.5 Clinical implications

Although the absolute risk of drug‐induced life‐threatening arrhythmias may be relatively low, given the number of patients treated with psychotherapeutic medications, even a small increase in the risk of SCD can have a substantial impact on public health.^35^ These AEs were more common in standard‐dose cases than in overdose cases, and approximately 8% of these AEs were severe enough to cause death or permanent disability. Approximately, 85% of the AEs reported were spontaneous reports by health care professionals, including physicians and pharmacists, and fewer than 12% were reported in clinical trials. Efficacy and safety must always be evaluated to find the best psychiatric medication for the patient. Investigation of AE reports using the JADER database is clinically important because it can clarify the risk of QT prolongation and TdP posed by drugs used according to the approved dosage and administration in Japan.

### 4.6 Study limitations

The main limitation of this study is that the database used is a spontaneous report database: (1) The number of all patients who received each drug could not be ascertained from this database, and the frequency of occurrence of each AE type by patient could not be calculated. (2) The number of reports varies depending on the level of interest of the reporter. (3) There are many missing values for each of the reported items. (4) The JADER database only provides information on the reason for drug use, response (e.g., dose reduction, discontinuation), and outcome; it does not provide details on the individual clinical course. (5) The TdP/VT/VF cases included in this study could not accurately capture the profile of patients who died before being transported to the hospital. Therefore, this study is likely to underestimate the true number of TdP/VT/VF patients. (6) Inadequate risk stratification methods, the possibility that the true situation was underestimated because the prescribing physicians were often not cardiologists, and the possibility that AEs were missed or discovered later without prior knowledge are other limitations. Finally, these data include several reporting biases, such as the Weber effect, notoriety bias, and the ripple effect, because it is a spontaneous AE reporting database.

## 5 CONCLUSIONS

This study using the JADER database showed that typical antipsychotics, antidepressants and antidementia drugs were associated with life‐threatening ventricular arrhythmias. Events were more frequent among women and elderly individuals; trazodone, sulpiride, and donepezil had high aRORs. Although QT prolongation is a risk factor for TdP, factors other than drugs, such as age, may be involved in the development of life‐threatening ventricular arrhythmias.

## FUNDING INFORMATION

This research received no grants from any funding agency in the public, commercial or not‐for‐profit sector.

## CONFLICT OF INTEREST STATEMENT

T.S. has received lecture fees from AstraZeneca, Bristol‐Myers Squibb, and Daichi‐Sankyo and has received grant support from Ono Pharmaceutical. S.Y. and M.H. have no conflicts of interest to declare.

## ETHICS STATEMENT

Information on all cases retrieved from the JADER database of individual case safety reports is deidentified, and ethical approval was not needed.

## PATIENT CONSENT STATEMENT

Not applicable.

## CLINICAL TRIAL REGISTRATION

Not applicable.

## Supporting information


Table S1.
