# Supplementary material for: Psychotherapeutic drug‐induced life‐threatening arrhythmias: A retrospective analysis using the Japanese adverse drug event report database
Source: J Arrhythm. 2023 Oct 3;39(6):928–36. doi: 10.1002/joa3.12936 (PMC10692844; doi:10.1002/joa3.12936)
Supplement: Supplementary file 1 — Table S1. [file JOA3-39-928-s001.docx]

Table S1. Underlying disease items used for adjustment.

Acute heart failure

Angina pectoris

Arrhythmia

Atrial fibrillation

Atrial flutter

Atrioventricular block

Bradyarrhythmia

Bradycardia

Cardiac defibrillation

Cardiac disorder

Cardiac dysfunction

Cardiac pacemaker

Cardiac sarcoidosis

Chronic heart failure

Chronic kidney disease

Complete atrioventricular block

Congenital long QT syndrome

Congestive cardiomyopathy

Congestive heart failure

Coronary sclerosis

Electrocardiographic QT prolongation

Essential hypertension

Heart failure

Hypertension

Hyperthyroidism

Left bundle branch block

Left ventricular hypertrophy

Long QT syndrome

Mitral regurgitation

Mitral stenosis

Myocardial infarction

Myocardial ischemia

Sinus node dysfunction

Stress cardiomyopathy

Supraventricular arrhythmia

Supraventricular premature contraction

Unstable angina

Ventricular fibrillation

Ventricular tachycardia
